# On Sleep and Sleeplessness

**Published:** 1849-07-01

**Authors:** 


Art. II.—*Sleeplessness in Disease—Lecture LXIX. in the Clinical Lectures on the Practice of Medicine.* By Robert J. Graves, M.D., M.R.I.A., &c., &c. Second Edition, Edited by J. Moore Nelligan, M.D., M.R.I.A. 2 vols. Dublin, 1848.

Sleep is a perpetual phenomenon in the twilight between life and death. That one-half or one-third of our existence should be passed in sleep, is only not surprising because it is familiar to us; but that our minds, furnished and stored as they are, with innumerable thoughts, increasing knowledge, and living recollections of things long past or late, should be suspended for such long and repeated intervals in the deepest oblivion, is indeed a startling fact, if we regard ourselves in the light of rational beings instead of mere animals. The most exciting affairs give way to sleep. The battle ceases to roar as the shades of night settle on the sickening strife—hostile armies consent to lay aside their animosity and defer the decision of the contest, until mutually re-invigorated with something like repose. And the eagerness with which we look for sleep, and the disappointment we feel at not being able to secure it—as if to become nothing for a time were a blessing or a benefit, and to lie like a living corpse, helpless and exposed to the chances of accident—the peril of assassination, fire, robbery, or violation—were a state to be sought for and coveted as a condition of the gift of life, most sorely to be regretted if missed or lost;—this is indeed a startling fact, and a supernatural item in the mystery of our creation!

If the nature of our being is essentially spiritual, how is it possible to suppose it can in its spirituality ever be so fatigued as to require sleep in order to recover from exhaustion? Nay, how can a spiritual being ever be exhausted or capable of going asleep? Or if it be thus by virtue of its union with the flesh, in what manner can the flesh sleep, and the spirit remain active and hold itself apart, while its weaker messmate, the body, is sunk in the slumbers of forgetfulness? Congestion of the brain by carbonized blood may be adduced as an explanation; and so may darkness, and expended irritability of the nervous tissue, and a law of periodicity, and the horizontal posture, and many other physiological terms and phrases in common use, serve the purpose of seeming to explain what has hitherto proved inexplicable. This is only one of the many faults in the stale system of hackneyed philosophy, so long in vogue, of explaining away facts by means of a supposititious cause, which is equally as inexplicable and unintelligible as the fact itself. And then, as to the nature of dreams—are they not always in accordance with the physical and moral conditions of the individual, be they morbid or healthy, foolish or rational, modest or depraved! Youth dreams on scenes of pleasure and activity—he swims, flies, acts, and enjoys himself; valetudinarians dream of nothing but what is vexatious and disagreeable to their senses; old men dream of the past; the traveller lying down to rest in the desert, is at home with his wife and young ones as soon as he shuts his eyes: merchants dream of cupons and the fluctuating price of bullion; martyred saints of the crowns of glory laid up for them in everlasting bliss; and the criminal under sentence of death, and condemned to be hanged on the morrow, dreams of green fields, happy faces, and the delights of his childhood. Who shall explain this? or, if explained, it could only be a mysterious explanation of what is already so transcendentally mystical. Sleeping and waking, life and death, action and repose, virtue and vice, weal and woe, respiration and circulation, food and nourishment, birth, growth, and decay—all are wonderful, and, with marvellous impenetrability, obstinately conspire to bid defiance to the keenest insight, the sharpest sagacity, and the closest reasoning.

Plutarch, endeavouring to show how we may know if we have acquired the habit and perfection of virtue, points out twelve ways, one of which is that of dreams. If even in your dreams, he says, you have no idea but what is right and proper; or if when others come upon you, you find that even in your sleep you struggle like a valiant soldier to resist them as strenuously as if you were awake, it is a sign that virtue is deeply rooted in you; because not only the will, but even the imagination and senses are made subject to reason. This agrees with the explanation of some authors on the passage of St. Paul in Thessalonians (1 Thess. v. 10), that not only when we are awake, but even when asleep, our thoughts should always be flowing in the same current and directed towards the same end. So Aristotle says, that when a man is master of any art or trade to perfection, he can so easily put it into practice, that he has no occasion to wait and think how he shall set about it; and all philosophers hold that habit appears not in the actions performed with reflection and care, but in those done upon the spur of the moment without having any leisure allowed us for deliberation and circumspection. It is spontaneous behaviour that evinces the instinctive habit of the mind and heart. Hence it follows, that we are as responsible for our dreams as we are for any one of our waking thoughts, just as much so, as, we are told, we shall be for every idle word at the great tribunal. For the nature of our dreams is determined by the ηθος or tone of mind which we cultivate, indulge in, or abandon ourselves to; so that, whether sleeping, waking, or dreaming, a man's life is nothing else than a dramatic personification of his innate or acquired ideas, or, in other words, it is his mind in *deed.* The phantoms of the night reappear as the motives of his conduct in the day, in the same manner as the deeds of the past day rise up in judgment for or against him in the fitful visions of the night. Accordingly the masters of the spiritual life have taken occasion to prescribe rules to their disciples concerning the proper manner of composing themselves to sleep, their mode of sleeping, and the manner of their dress, the kind of thoughts with which they should entertain themselves as they close their eyes, what they should do if they could not sleep, and how they should recollect themselves upon first awaking in the morning; for they justly concluded, that in no moment of his life should a good man ever neglect himself, or permit his great enemy death to steal upon him unawares.

Every one, when asleep, has his own world; but when awake, he lives in the world of others. And yet this world of others is but a particular apparition seen in a peculiar light by each individual separately at the same time, equally as much so when he is awake as when he dreams. The external world of nature is evermore the same,—its winter and summer, storm and sunshine, flood and drought, earthquake and calm, seed-time and harvest-time, always remain the same; but our thoughts, or modes of thinking, make it appear altogether various at its different epochs, centuries of epochs, and ages of centuries, diversified throughout by hosts and hosts of thoughts, each with different views, tending to one great centre of self-interest, continual action, and a progressive development of events. Hence comes the gorgeous pageant that emblazons the page of history, glittering in solemn procession, as with measured step it treads the crowded track, and exhibits in review the several actors in their full costume of mind and person. Each figure stands out distinctly by itself from the rich background of the vast ideal, and yet forms a part together with the brilliant multitude of images, grouped into masses of the most exquisitely pleasing colours of light and shade. Dreaming is a continued whole running parallel with the waking world, but without interfering with it. When awake, we scarcely remember the dreams of our deepest sleep, whereas often in the dreams of to-day we remember those of yesterday. In dreams, we know persons, places, &c., that we never knew when awake, which reappear, however, in our succeeding dreams; nay, persons who are known to us appear to us in our dreams otherwise than they are, but in one dream the same as in another.

There is something so evanescent and so fleeting in our existence, that the whole is but a dream—a fugitive vision, a glowing illusion—that vanishes as we approach it, or breaks into a thousand fantastic shapes while we stop to behold it. Friends come, go, and disappear without leaving a trace of their footsteps behind them; their voices are still ringing within our ears when their spirit is far away, and their forms are mouldering in the grave. Our days fly like the bird in the air, or the vapour that dissolves in the first beams of the morning. Kingdoms rise and fall within sight of home; the earth smells rank with the carnage of the battle-field; and the clinking of gold at our feet makes one shudder with horror as the eye ranges along the lurid horizon and discerns the distant tempest lowering with famine and despair. And time rolls forward with a velocity that it is frightful to contemplate—hurling the bodies and the souls of men adown the gulf of destruction with an irresistible, appalling, and majestic force! But away with it!—there is no such thing. It is a dream: let us awake.

Beyond the range of our senses is that of the spirit; our first perceptions, acquired ideas, judgments, deductions, principles, and the practical and intellectual consequences following from thence—everything, in fact, of which we have any consciousness and certainty. Nor is it probable that the multitude of thoughts that throng our minds should, according to the fanciful expression of the poet, be "lost in thinking and dissolved in air." Such a conclusion is out of all congruity with the Divine mind, which works by means for an end, and this end is brought about by means of thinking—our thoughts are eternal. But what is the substance that thinks and knows?—fibrous neurine, tubular neurine, nervous fluid, vital principle?—what? Spiritualists call it the soul, and say that it is a substance entirely different from the body—without figure, colour, weight, measure, or divisibility—a being which eludes every effort of the imagination to conceive it, and defies every mode of expression to describe it. Who has ever seen it? And, as to those who conceive it, no one can tell us what it is. Materialists, on the contrary, maintain that the soul is a mere cipher, and that thought is nothing more than the result of a perfect co-adaptation of certain particles of matter in the living frame, inasmuch as, they say, no one has ever met with a sound mind except in a sound body. Setting aside the vicious circle of this mode of reasoning, we call upon them to define, first of all, what is the sound condition of body requisite? Be this, however, as it may, it is evident, that the perfect soul must allow the perfect body to go asleep once at least every twenty-four hours, or otherwise the perfect body can no longer serve the perfect soul, account for it as we please.

Truth lies beyond the columns of Hercules. The mariner's compass of science is as wide as the circumference of the universe. Is it possible to go further than this? And yet, notwithstanding, the truth lies still much further off. The needle traverses every point marked down upon the card of infinitude, and returns to the point from whence it started, still pointing to what is infinite; nor is there any one point at which we can stop the wavering magnet and cry out, "Hold, enough!" See, there stands fixed before us the load-star of the world! No, by our halidom, it is not fixed there —it is only a star among many stars; and if it appear in magnitude much larger than the rest, it is only because it is infinitely nearer than the others, which are infinitely further off. Truth is a mighty ocean, whose horizon is as immeasurable as its depth is unfathomable. Confined, like a pebble, to a single spot on the surface of this terrestrial globe, which is itself a spot, or particle in the vast abyss of the firmament, we have no theodolite by which to find the level of space, nor any arithmetical tablet ready at hand for working out a logarithm, for the purpose of taking the altitude of life. We gaze upon the ocean of science, and, having no boat withal, however frail, to embark upon its boundless waters, we stand upon the shore, and content ourselves with counting the unceasing waves that chace each other in succession and break along the beach.

It is the same with all our inquiries; and the ablest psychologist must admit that it is beyond his power to explain the nature of sleep. As a portion of the invisible world it attracts our attention and pricks our curiosity. Theories have been broached, conjectures hazarded, and opinions entertained respecting it; nevertheless, the best works on physiology offer us nothing more satisfactory than probable opinions, curious conjectures, and interesting theories, while pretending to solve a problem which still remains, as it has hitherto remained—the monitor of the end of all things—the daily rehearsal of our death:—*Memento novissima tua, sic non in æternum peribis.*

There are some curious instances on record of sleeping and waking. In Turkey, if a person happens to fall asleep in the neighbourhood of a poppy-field, and the wind blows over towards him, he becomes gradually narcotized, and would die if the country people, who are well acquainted with the circumstance, did not bring him to the next well or stream, and empty pitcher after pitcher on his face and body. Dr. Oppenheim, during his residence in Turkey, owed his life to this simple and efficacious treatment. Dr. Graves, from whom this anecdote is quoted, also reports the case of a gentleman, thirty years of age, who, from long continued sleeplessness, was reduced to a complete living skeleton, unable to stand on his legs. It was partly owing to disease, but chiefly to the abuse of mercury and opium, until, at last, unable to pursue his business, he sank into abject poverty and woe. Dr. Reid mentions a friend of his, who, whenever anything occurred to distress him, soon became drowsy and fell asleep. A fellow-student, also, at Edinburgh, upon hearing suddenly of the unexpected death of a near relative, threw himself on his bed, and almost instantaneously, amidst the glare of noon-day, sunk into a profound slumber. Another person, reading aloud to one of his dearest friends stretched on his death-bed, fell fast asleep, and, with the book still in his hand, went on reading, utterly unconscious of what he was uttering. Hippocrates relates the instance of a young man who fell asleep on his back in a tent, after having drunk too freely of pure wine, when a serpent crawled in at his mouth. Awaking with a start, he gnashed his teeth, and bit off a portion of the reptile, as he swallowed the rest; upon which he fell into convulsions and died. He likewise refers to the case of a priest, subject to attacks of the gout, followed by a lethargy, or unusually prolonged sleep. A woman at Henault slept seventeen or eighteen hours a day for fifteen years. Another is recorded to have slept once for forty days. A man, twenty-five years of age, at Tinsbury, near Bath, once slept for a month, and in two years he slept again for seventeen days. Dr. Macnish mentions a woman who spent three-fourths of her life in sleep; and Dr. Elliotson, who has collected several instances of this sort, quotes the case of a young lady who slept for six weeks, and recovered. Herodotus, in "Melpomene," alludes incredulously to a race of the Scythians or Tartars, in the extreme north, who were reported to sleep away six months of the year. Two young gentlemen, says Dr. Graves, college students, went to bed in perfect health the night previous to their examination; they slept soundly; the elder one rose early in the morning, and left his younger brother in bed still asleep; he remained so for two hours more, having slept altogether for more than ten hours, when he awoke in a state of complete insanity. The same author, likewise, relates the case of a gentleman who fell asleep with his head resting on his hands, folded together before him on the table after dinner. On awakening, one arm was paralyzed, and remained paralytic to the day of his death, which followed not long afterwards. The celebrated

General Elliot, Frederic the Great, and John Hunter, seldom slept more than four or five hours in the twenty-four. Dr. Macnish mentions a lady in perfect health who never slept more than three or four hours in the twenty-four, and then only half an hour at a time. General Pichegru, according to Sir Gilbert Blane, had only one hour's sleep in the same space of time for a whole year. The venerable St. Augustine, of Hippo, prudently divided his hours into three parts—eight he devoted to sleep, eight to recreation, and eight to converse with the world. De Moivre slept twenty hours out of the twenty-four; Quin, the celebrated player, could at his pleasure slumber twenty-four hours in succession; and Dr. Reid could, when he liked, take as much food and as much sleep as would serve him for a couple of days. Richerand gives the instance of a man in the hospital of St. Louis, who slept five-sixths of the day, and awoke only to appease the cravings of a voracious appetite. Attila, the scourge of God, died on his bridal night from the bursting of a bloodvessel in his sleep, and his trembling bride was found sitting by his bed-side hiding her face in her veil, and lamenting her own danger as well as the death of the king. Theodosius, falling asleep in the morning watch of his last great battle, saw in his dreams an apparition that assured him of the victory over his desperate foe, Eugenius, and the issue of the forthcoming day verified or coincided with this strange presentiment. The dauphin, son of the unfortunate Louis XVI., the descendant of the sovereigns of France and Navarre, shut up in a loathsome nook, with a hole in the wall through which his scanty rations were thrust, was killed by the want of sleep. His feverish temples were scarcely laid upon his pallet, when a stern voice peeled round the walls—"*Capet, où es tu ? dors tu ?*" By a refinement of cruelty of this description, his ductile and confiding spirit, drawn out to the last gasp, silently gave up the ghost on the 8th of June, in his 10th year, 1795. The famous St Dominic, of Calarueza, in Spain, never reposed except on the floor or the bare boards, which served him for a bed; St. Bonaventura, one of the first Franciscans, made use of a common stone of some size, instead of a pillow; and St. Peter, of Alcantara, slept but one hour and a half in the twenty-four hours for forty years together, either kneeling or standing, with his head leaning aside on a little piece of wood fastened for that purpose in the wall. He usually ate but once in three days, yet he lived to be old, though his body was so attenuated and weak that it seemed to be composed of the roots of trees, and his skin so parched that it resembled the dry bark of a tree rather than flesh. People may sleep in all sorts of postures. According to Mr. Wilkinson, the ancient Egyptians, who, as every body knows, shaved their scalps, slept with their heads resting on an iron prong, like that of a pitchfork, welted with something soft. This they did for the sake of keeping their heads cool, which they supposed strengthened their wits. The postillion will sleep on horseback, and the sentinel at his post. An entire battalion of infantry have been known to sleep on the march. It is about three or four o'clock in the morning that this propensity to sleep is the most overpowering—the moment seized upon by troops for driving in the enemy's out-posts, and taking the bivouac by surprise. Maniacs are reported, particularly in the eastern hemisphere, to become furiously vigilant during the full of the moon, more especially when the deteriorating ray of its polarized light is permitted to fall into their apartment—hence the name *lunatics*. Sleeping directly in the moon's rays is said to be at all times prejudicial. There certainly is a greater proneness to disease during sleep than in the waking state; for those who pass the night in the *Compagna di Roma* inevitably become infected with its noxious air, while travellers who go through without stopping escape the miasma. Intense cold induces sleep, and they who perish in the snow sleep on till they sleep the sleep of death.

All nature sleeps. Plants sleep in the winter, reptiles and some animals hybernate, and the earth sleeps as she wheels into darkness from west to east. Children sleep a great deal—infants much more, but with irregularity; while the old man scarcely slumbers at all, watching as it were his end approaching. Even the fœtus sleeps in a sort of indeterminate manner; and public affairs have their grand alternations of action and repose. The heart sleeps between every beat sixty times in the minute, and so do all the other organs with more or less regularity. Everything that has life must sleep or else it dies, which is a marvel beyond our comprehension.

People may sleep too little or too much, too early or too late. Overmuch sleep conduces to obesity, torpor of the general functions, congestions of the chief viscera, more especially of the head, endangering attacks of apoplexy and death. It is the *bon vivant* who is disposed to somnolency—a dose in his easy chair after dinner, and an inclination to nod over the newspaper or during a prolonged discourse. The well-nourished require more sleep than the lean, and the phlegmatic more than the irritable. But in the present day somnolent obesity is a rare phenomenon. The evil that we have to complain of is an incapability of sleeping enough. There is no fixed duration for sleep. The world roars around us like a torrent of events. Everything is rapid; and we are whirled with velocity in the midst of a vortex, as vast as it is incessant. Repose there is none; and instead of sleeping on a pillow of down, we stand continually on the tiptoe of expectation, awaiting the coming on of to-morrow, big as it were with the doom of some great hereafter. It is impossible to sleep—nay, it is scarcely possible to survive. This morbid excitement, fictitious let it be, is in reality the pregnant source of a large family of ailments, of which mania is neither the youngest nor the most insignificant child.

Opium in some of its forms is supposed to be the chief ingredient with which we drug the posset of repose. But this is by no means the case. For, in the kind of vigilance just alluded to, opium in its most concentrated form is most frequently useless. Sometimes it increases the vigilance, and, instead of soothing, excites the nerves to ecstasy, in which the patient hears, sees, and converses with phantasms that have no existence save on the irritated expansion of his diseased retina. In the sleeplessness of the insane, this is particularly true; opiates very often exert no influence over their moon-stricken fancies. They must be watched with the utmost caution, and sleep must be coaxed and solicited by means of remedies that have nothing of the narcotic principle in them. Mesmeric passes (be mesmerism what it may) will induce sleep when nothing else will, and it is a kind of sleep altogether exempt from the subsequent distress arising from the use of an opiate. Allowing the patient to get up and wash himself and walk about his room, making his bed afresh, giving him a glass of cold water, or bitter ale, or wine-and-water, will often succeed in procuring refreshing sleep when all other means have failed. These are practical points calling for experienced judgment in their application.

The condition of the eye will generally afford a correct criterion for determining the kind of narcotic or sedative to be prescribed. For, when the eye is bloodshot and the pupil contracted, opium is rarely effectual, the vascularity of the eyeball implying that the cerebral circulation, especially that of the meninges, is already too much injected. Neither would stimulants of any sort be serviceable at such times. Hyoscyamus, conium, and aconite are more to be relied upon; or if opium be prescribed, it should be conjoined with tartar emetic, salines, and nitre, or with ipecacuanha, as in the Dover's Powder. With antimony, it will occasionally succeed, although it would fail if given by itself. The success of this combination of opium, sudorifics, and nephritics, arises most likely from their acting on the entire surface of the skin, and thus relieving the internal organs, and allowing the opiate in the mean time to allay the unsettled mobility of the nerves. Dr. Graves is convinced that antimony exerts a decided narcotic influence on the system. Opium, likewise, will often act favourably when given with some of the alkalies; but its mildest, if not its surest, way of narcotising is that pointed out by Dupuytren, when applied to the orifices of the mucous membranes.

On the contrary, when the conjunctiva is pale, and the pupil dilated, stimulants prove the most certain narcotics; for these symptoms indicate the sleeplessness of inanition and the necessity for support. In this stage, which may forerun mania in its most troublesome form, large and repeated doses of pure opium have been given with the happiest results. Dr. Crawford found opium, gradually increased to large and frequently repeated doses, so as to produce sleep, the best remedy. It is a condition which follows the loss of blood, as from venesection or hæmorrhage, when hyoscyamus with camphor is so beneficial. In those instances of watchfulness which are frequently observed towards the close of acute diseases, it is always necessary to repeat the opiate for some time after the first symptoms have been checked. There is no fear of giving successive doses, lest the patient should become accustomed to them, and a bad habit be generated, for the rapid convalescence and renewed health, which are wonderfully promoted by securing a sound and refreshing sleep, will soon enable him to dispense with the use of an opiate. In these cases, the upward circulation is too feeble, and the *vis a tergo* must be increased and sustained. The same practice is likewise pre-eminently useful in the first stage of delirium tremens, in its congestive onset, before the subacute inflammation and milky effusion have ensued. The irritability, watchfulness, and phantasmagoria of this peculiar malady are best treated by a combination of bitters, alkalies, and opiates; or else with tonics (even steel), local or general depletion, and opiates, at the same time.

Nothing, however, relieves the vigilance of old age; nor should opium ever be tried, since the dose that is sufficient to procure sleep may end in death. Persons have been destroyed by a single dose of a third of a grain of muriate of morphia, and two or three from a grain, although they may have recently taken doses of half a grain, or even a grain, with little effect. Yet a grain of muriate of morphia may be taken every hour for forty-eight hours without any effect. Sometimes, without dying, persons remain a long time asleep after soporifics. Dr. Macnish, quoted by Dr. Elliotson, refers to a child, near Lymington, that was thus sent asleep for three weeks. The proper remedy, and the particular dose, require the tact and management of one engaged in extensive practice, for an experienced hint is no rule to a novice. Sometimes hot sponges applied to the head will cause sleep, and sometimes cold; but cold will awaken the sleeper, and so will heat. These are the difficulties of medicine that render it an *art*, instead of constituting it a *science*, and make the expert practitioner so invaluable with the public. Successful practice is often empirical.

The power of the will may obtain or dissipate sleep. Some persons have the power of willing themselves asleep as soon as they lie down. Mr. Binns, in his "Anatomy of Sleep," gives directions to this effect; and there is no doubt that the habit of doing so may be easily acquired.

Much will depend upon the time and manner of administering a narcotic. The general practice is to give it at the usual hour of bed-time; but this may do nothing more than tease the patient with a soporific that does not produce sleep. A more delicate mode of proceeding is that of denoting the particular hour when the vigil relapses into a brief slumber, and to order the narcotic to be taken an hour before this wished-for moment arrives. Thus the remedy will coincide with the course of the natural changes, and begin to operate just as the habitual slumber is coming on. In hectic, this period is about four in the morning; in ague, it is generally in the afternoon; in rheumatism, at the evening twilight; and in consumption, about midnight. In putrid fevers, the prostration of strength shows itself almost always in the morning; and petechiæ, as well as gangrene, those fatal signs of debility, break out and proceed during sleep in the night.

Diet likewise may be converted into a narcotic sedative. Tea or coffee will produce sleep when the brain is plethoric, as surely as it will give rise to the most vexatious vigilance and nervous irritability when the brain is already exhausted. Thus, men who have studied hard and lived too low will fall asleep after a good dinner, while those who have taken their wine and lived too richly will not be able to shut their eyes until they have been served with a cup of pure coffee first and their green tea afterwards—the tea and the coffee allaying the upward circulation, and acting as an immediate sedative on the nerves. Bleeding or digitalis would of course answer the purpose just as well.

A sleepless night cannot be recovered from by any subsequent siesta snatched out of the business of the day. We must wait for the following night, go to bed early, and sleep soundly, if we hope to awake refreshed the next morning. Nor can the exhaustion from the want of sleep be relieved from stimulants, either in the shape of food or medicine, although the late hours of the modern world, which induce a perpetual lassitude both of mind and body, are alleged as one of the chief reasons, if not of the many poor excuses, for indulging in wine and hot condiments. There is a form of conditional drunkenness to which people in good society are addicted, without being aware of it, that produces effects quite as pernicious as the dram-drinking among the lower orders—we mean the free use of wine at the dinner-table. Such persons, without suffering in appearance or losing flesh, get into a chronic state of disturbed health, manifested by impaired digestion and irritable nerves. Deprived of his usual modicum, the gentleman is unable to sleep, and becomes, in a certain degree, delirious, unless allowed to return to his ordinary habits. There is likewise a chronic sleeplessness, chiefly among the better classes, where individuals suffer from an almost total want of rest for months together, without any loss of flesh or visible impairment of the constitution. Such cases get well of themselves, after a shorter or longer period, and do not require any medical treatment.

The evil consequences of not sleeping enough are clearly manifested in the features, which become pale, lank, and sharp; the eye cold, blanched, and watery; the hair shabby, straight, and long; the deportment wan; and the feelings languid. The palms of the hands are hot, the lips dry and peeling, and the utterance feeble or tremulous, while a low fever feeds upon the vitals. If the want of sleep is voluntary, as in the pursuit of some necessary or interesting occupation, or in consequence of fashionable engagements, it saps the strength at an early period; men become old at thirty-five or forty, and women, wasted in their prime, suffer from difficult childbirth, or die in consequence of it. A metropolitan life is most baneful in this respect, and may be considered as limiting the average of longevity to forty-five or fifty years.

Those who go to bed late rise late, and early risers are for the most part forced to retire equally early. Students, who require more sleep than others, usually rise too early and sit up too late. Modern fine children, who are taught to mimic their elders, are exotics, flowering in an artificial atmosphere, but withering without fruit long before the morning of their days has passed over their debilitated heads. There is no compensation for the loss of health. Nor learning, nor fame, nor money, nor power, is equivalent to an elastic, vigorous constitution; nor are the lesser virtues, usually styled accomplishments, pleasing and graceful as they may be, of any value in comparison with the decrepit nerves and the still more decrepit morals with which they have been purchased.

The older physicians paid much closer attention to this inquiry than modern physicians and physiologists are accustomed to do. Hippocrates long ago pointed out the importance of denoting the kind of sleep, the nature of the dreams, and the particular posture of the sleeper in bed, as an accessory means of forming a correct diagnosis of his disease. Allowing for some puerilities peculiar to the remote epoch in which he flourished, the fourth book of his treatise, entitled "Regimen," is a much more practical essay on this subject than anything else of the sort that has as yet been put forth by later pathologists.

Although Dr. Marshall Hall observes that sleep is a cerebral affection, and that the spinal and ganglionic systems never sleep, yet our notions are, we cannot help avowing, somewhat different from his upon this subject. It seems to us, that the power of sleeping is much more intimately connected with the medulla oblongata and the spinal cord than with the cerebrum and superior portions of the hemispheres. The whole nervous system is a unit or entity in its essence, compounded of distinct parts entirely united in their different, but by no means separate functions. The intellectual powers would seem to belong to the cerebra, perhaps to the cortical substance, or grey matter alone, as some suppose; the several senses would appear to have their origin or root at the base of the brain; the faculties of the mere animal organs are apparently governed by the cerebellum, while those of locomotion, speech, respiration, &c., are evidently connected with the pons varolii, the medulla oblongata, and the spinal cord. Now sleep is an absolute suspension of sense and motion; but then these two functions, or sets of functions, are under the rule of the spinal cord; so that the spinal cord would seem to be the immediate locality, if not the true centre, both of rest and action, progression and repose, locomotion and inertion, or, in other words, of waking and sleeping.

There are several physiological as well as pathological phenomena confirmative of this view of the case. It has been proved, independently of Dr. Graves' personal testimony to the fact, that the pupil of the eye is closed almost to a point, not larger than a pinhole, during sleep; that the eyeball likewise is turned upwards; and that the upper eyelid falls down. These phenomena are also the symptoms set down as indicating disturbance at the base of the brain among the diseases of that organ. For a very contracted pupil is almost always a fatal sign, by showing loss of power at the origin of the respiratory nerves, whether from effusion, or injury, or natural dissolution. The turning upwards of the eyeball is a common symptom of convulsive affections proceeding from spinal irritation, or mechanical mischief of that part. And the drooping of the eyelid is indicative of incipient paralysis, or loss of power, in the cord and medulla oblongata, often forerunning paraplegia, loss of speech, and failure of the locomotive power in general. These signs, which are, physiologically speaking, in one sense the phenomena of ordinary sleep, become, in another, leading symptoms in the natural history of disease of the brain; and both the symptoms, on the one hand, and the phenomena, on the other, point directly to one part of the grand system of nerves—namely, the medulla oblongata, and the spinal cord.

That sleep should be less connected with the cerebrum than with the cord is not so unlikely, if we consider the nature of each part of the nervous system respectively; for we shall be enabled to remark, that sleep, which is the suspension of action, belongs to the spinal portion rather than to the brain or cerebrum, which, as the focus of will and intelligence, only exerts itself in governing the rest of the frame, and ceases from exertion as soon as the rest of its parts cease to require governing. Disturbance of the superior hemispheres will of course hinder the cord from sleeping quite as effectually as irritation of any other part of the body would do; but we mean that, in health, sleep belongs to the cord rather than to the brain. Vigilance is one of the most vexatious symptoms of spinal disease—of spinal exhaustion from venery, or excessive pedestrianism—of spinal irritation produced by a sort of reflex action from acid indigestion, accompanied with subsultus and cramps of the legs, and of profuse diarrhœa, exhausting the spinal cord so greatly as to give rise to incurable paraplegia. All these signs, symptoms, and phenomena lead to one and the same conclusion—that the spinal cord rather than brain is chiefly concerned in sleep and sleeplessness.

There is another chain of proofs which conducts us to the same end—namely, those drawn from the action of narcotic medicines. When given for the purpose of overcoming sheer sleeplessness, apart from the intervention of any other disease or disturbing agency among the various other functions, they act more decidedly when applied to the orifice of the inferior mucous membrane, or used externally along the spine, than when given by the mouth. This effect is obviously spinal.

The mesmeric sleep has been so unscientifically handled by profane experimenters, that a legitimate inquiry into its real nature is almost interdicted pursuant to a decree of the high court of medicine. No one, however, who has once witnessed its phenomenon, can pretend to say that it does not exist; nor are we, on our part, prepared to discard its facts as *res infimæ et nugatoriæ*, or to turn our backs upon an inquiry which is, at present, we own, beyond our ken. There can be no doubt that the mesmeric sleep is real, and that it differs from ordinary sleep in its intensity as well as in the automatic movements of the spinal system, together with the trans-luminous consciousness of the dormant faculty of thinking. It is evident that the sensorium is in this state made conscious of the operation of functions, morbid or healthy, which in its ordinary or rational waking condition it is not alive to, and that the nerves communicate to the brain impressions of inward sensations which are not reported or conveyed thither under ordinary circumstances. The peripheral nerves are strangely excited, the sense of pain arising from injury or disease is diminished or annulled, and disease itself suspended or removed. The *quo modo* of this state of things is the very point in question, and it remains for the orthodox physiologists who deny the matter to bear the burden of the proof; although thus much is incontestible, that the medulla oblongata and spinal cord play a much more prominent part in these curious manifestations of simple organic life than the mesmerists themselves, as far as we are able to understand them, seemed disposed to allow.

The invisible agency of thought existing not except by the visible agency of the brain, constitutes both the popular philosophy of ghosts or spirits, and the medical philosophy of soul and body; added to which, the world of dreams has rendered the philosopher little less fanciful and incoherent than the uninitiated vulgar, who, in his turn, seems, at times, to be scarcely more intelligible than the wisest philosopher.

